# Supplementary material for: IC3 protocol: a longitudinal observational study of cognition after stroke using novel digital health technology
Source: BMJ Open. 2023 Nov 24;13(11):e076653. doi: 10.1136/bmjopen-2023-076653 (PMC10679983; doi:10.1136/bmjopen-2023-076653)
Supplement: Supplementary data [file bmjopen-2023-076653supp001.pdf]

Supplementary Table 1 - Inclusion and Exclusion Criteria for Patients

Inclusion Criteria	*Exclusion Criteria*
○ Aged > 18<br>○ Evidence of confirmed stroke (not limited to first stroke)<br>○ Ability to concentrate for 15 minutes at a time to engage with cognitive testing	*For the main study:*<br>○ Pre-stroke diagnosis of dementia<br>○ Severe visuo-spatial problems<br>○ Fatigue limiting engagement in the tasks beyond 15 minutes<br>○ Active severe mental health diagnosis (e.g. severe depression or severe anxiety)<br>○ Severe hearing impairment in the presence of reading comprehension impairment resulting in inability to follow task instructions
	For the imaging sub-study:<br>○ Pregnancy<br>○ Ferromagnetic implants<br>○ Claustrophobia
