# Supplementary material for: IC3 protocol: a longitudinal observational study of cognition after stroke using novel digital health technology
Source: BMJ Open. 2023 Nov 24;13(11):e076653. doi: 10.1136/bmjopen-2023-076653 (PMC10679983; doi:10.1136/bmjopen-2023-076653)
Supplement: Author's manuscript [file bmjopen-2023-076653.draft_revisions.pdf]

BMJ Open is committed to open peer review. As part of this commitment we make the peer review history of every article we publish publicly available.

When an article is published we post the peer reviewers' comments and the authors' responses online. We also post the versions of the paper that were used during peer review. These are the versions that the peer review comments apply to.

The versions of the paper that follow are the versions that were submitted during the peer review process. They are not the versions of record or the final published versions. They should not be cited or distributed as the published version of this manuscript.

BMJ Open is an open access journal and the full, final, typeset and author-corrected version of record of the manuscript is available on our site with no access controls, subscription charges or pay-per-view fees (http://bmjopen.bmj.com).

If you have any questions on BMJ Open's open peer review process please email info.bmjopen@bmj.com

IC3 Protocol – A novel scalable method for monitoring cognition after stroke

Journal:	*BMJ Open*
Manuscript ID	bmjopen-2023-076653
Article Type:	Protocol
Date Submitted by the Author:	15-Jun-2023

Complete List of Authors:	Gruia, Dragos-Cristian; Imperial College London, Brain Sciences; Imperial College Healthcare NHS Trust<br>Trender, William; Imperial College London, Brain Sciences<br>Hellyer, Peter; King's College London, Centre for Neuroimaging Sciences, IoPPN<br>Banerjee, Soma; Imperial College Healthcare NHS Trust<br>Kwan, Joseph; Imperial College Healthcare NHS Trust<br>Zetterberg, Henrik; UCL, Department of Neurodegenerative Disease, Institute of Neurology; University of Gothenburg, Department of Psychiatry and Neurochemistry<br>Hampshire, Adam; Imperial College London, Brain Sciences<br>Geranmayeh, Fatemeh; Imperial College Healthcare NHS Trust; Imperial College London, Brain Sciences

Keywords:	Stroke < NEUROLOGY, Stroke medicine < INTERNAL MEDICINE, Magnetic resonance imaging < RADIOLOGY & IMAGING

SCHOLARONE™
Manuscripts

IC3 Protocol – A novel scalable method for monitoring cognition after stroke

Dragos C. Gruia[1,3], William Trender[1], Peter Hellyer[2], Soma Banerjee[1,3], Joseph Kwan[1,3], Henrik Zetterberg[4-9], Adam Hampshire[1], Fatemeh Geranmayeh[1,3]

1- Department of Brain Sciences, Imperial College London, London, W12 0NN, UK.
2- Centre for Neuroimaging Sciences, IoPPN, King's College London. SE5 8AF, London, UK.
3- Imperial College NHS Healthcare Trust, London W6 8RF, UK.
4- Department of Psychiatry and Neurochemistry, Institute of Neuroscience and Physiology, the Sahlgrenska Academy at the University of Gothenburg, Mölndal, Sweden
5- Department of Neurodegenerative Disease, UCL Institute of Neurology, Queen Square, London, UK
6- Clinical Neurochemistry Laboratory, Sahlgrenska University Hospital, Mölndal, Sweden
7- UK Dementia Research Institute at UCL, London, UK

For peer review only

8- Hong Kong Center for Neurodegenerative Diseases, Clear Water Bay, Hong Kong, China

9- Wisconsin Alzheimer's Disease Research Center, University of Wisconsin School of Medicine and Public Health, University of Wisconsin-Madison, Madison, WI, USA

Corresponding author:

Dr Fatemeh Geranmayeh

Clinical Language and Cognition group. Department of Brain Sciences. Imperial College London. London W12 0NN. UK

fatemeh.geranmayeh00@imperial.ac.uk

Keywords:

Cognitive testing, stroke, cerebrovascular disease, observational study, biomarkers

For peer review only

Abstract

Introduction. Stroke is a major cause of death and disability worldwide, frequently resulting in persistent cognitive deficits among survivors. These deficits negatively

impact recovery and therapy engagement, and their treatment is consistently rated as high priority by stakeholders and clinicians. Although clinical guidelines endorse cognitive screening for post-stroke management, there is currently no gold standard approach for identifying cognitive deficits after stroke, and clinical stroke services lack the capacity for long-term cognitive monitoring and care. Currently available assessment tools are either not stroke-specific, not in-depth or lack scalability, leading to heterogeneity in patient assessments. **Methods and Analysis.** To address these challenges, a cost-effective, scalable, and comprehensive screening tool is needed to provide a stroke-specific assessment of cognition. The current study presents such a novel digital tool, the Imperial Comprehensive Cognitive Assessment in Cerebrovascular Disease (IC3), designed to detect both domain-general and domain-specific cognitive deficits in patients after stroke with minimal input from a health professional. To ensure its reliability, we will utilise multiple validation approaches, and aim to recruit a large normative sample of age-, gender-, and education-matched UK-based controls. Moreover, the IC3 assessment will be integrated within a larger prospective observational longitudinal clinical trial, where post-stroke cognition will be examined in tandem with brain imaging and blood biomarkers to identify novel multimodal biomarkers of recovery after stroke. This study will enable deeper cognitive phenotyping of patients at a large scale, whilst identifying those with highest risk of progressive cognitive decline, as well as those with greatest potential for recovery. **Ethics and dissemination.** This study has been approval by South West - Frenchay Research Ethics Committee (IRAS 299333), and authorized by the UK's Health

For peer review only

Research Authority. **Study registration.** The study is registered as an observational trial under NCT05885295.

Strengths and limitations

- This study addresses post stroke cognitive deficits, a major clinical problem.
- Accurately predicting the trajectory of cognitive recovery is a largely unmet clinical and scientific need.
- We developed a novel digital screening tool called the Imperial Comprehensive Cognitive Assessment in Cerebrovascular Disease (IC3), that is not only cost-effective and scalable, but can also provide deep phenotyping of stroke-specific cognitive deficits with minimal input from a health professional.
- The IC3 tool will be used within a longitudinal observational study for identifying novel brain imaging and blood biomarkers of recovery after stroke.
- This study will enable large-scale cognitive profiling and monitoring of patients, improving our ability to identify those at the highest risk of progressive cognitive decline and those with the greatest potential for recovery.

Introduction and Rationale

For peer review only

Stroke is a leading cause of death and disability globally, with frequent cognitive sequalae affecting three-quarters of survivors [1]. The spectrum of cognitive deficits includes background 'vascular cognitive impairment' and more domain-specific deficits, such as aphasia which affects a third of patients [2]. Collectively, these impairments negatively impact post-stroke recovery and engagement with therapy [3]. Furthermore, there is a high failure rate of post-stroke cognitive rehabilitation trials, due to our poor understanding of individual patient-level mechanisms of post-stroke cognitive recovery and the ensuing inadequate stratification of both treatment responders and non-responders in the same clinical trials. It is therefore important to understand the factors that affect cognitive recovery post-stroke, a topic increasingly recognised by funders and stakeholders as a high priority research area [4, 5].

Although clinical guidelines have been recently updated to recommend cognitive screening for post-stroke management, there is currently no gold standard approach for identifying cognitive deficits after stroke [6]. Clinicians and researchers often select cognitive tests depending on preference, familiarity and availability, leaving little prospect for generalizability of their findings. Moreover, clinical stroke services do not have the capacity to provide long-term cognitive monitoring and care for stroke survivors who have unpredictable recovery trajectories [3, 7]. This challenge is further compounded by the lack of cost-effective cognitive tools suitable for long-term monitoring, and the reliance of such monitoring on resource-stretched healthcare professionals.

For peer review only

Availability of a cost-effective, reliable, and comprehensive screening tool that provides a stroke-specific deep phenotyping of cognition would be a game-changer. In the clinical setting, it would improve the detection rate and monitoring of cognitive deficits post stroke, and in the research setting it would facilitate much needed large-scale population-based mechanistic studies aimed at understanding the mechanisms of cognitive recovery. Here, we present such novel digital adaptive tool: The Imperial Comprehensive Cognitive Assessment in Cerebrovascular Disease (IC3). IC3 is a digital assessment designed to require minimal input from a clinician in detecting both domain-general and domain-specific cognitive deficits in patients after stroke. To ensure its reliability, several validation studies will be conducted against established clinical screening tools, and normative samples will be computed using representative age-, gender- and education-matched UK-based controls.

In line with recent efforts in identifying biomarkers of post-stroke recovery [8], the IC3 tool will be integrated within a larger prospective longitudinal clinical study, where post-stroke cognition will be examined in tandem with brain imaging and novel blood biomarkers. This study is entitled "Understanding factors affecting cognitive function in cerebrovascular disease" and will hereafter be referred to as the '*main study*'.

Methods and Design

For peer review only

Aims

1. Develop an adaptive, scalable, self-administered, digital comprehensive cognitive screening tool to detect cognitive impairments in patients with cerebrovascular disease. The IC3 will:
    I. Detect both domain-general (e.g. executive function) and domain-specific (e.g. aphasia, apraxia, neglect) deficits post-stroke [9].
    II. Allow monitoring to occur at scale, in a cost-efficient manner, as test administration can occur independent of trained professionals.
    III. Minimise the effects of neglect or aphasia on cognitive assessments.
    IV. Have high test-re-test reliability for repeated-testing longitudinally.
2. Validate the IC3 against commonly used cognitive screening tools.
3. Use IC3 in a cohort of stroke survivors to map the cognitive recovery trajectory over the course of the first year after stroke.
4. Identify novel biomarkers of cognitive outcome (at 1 year) and recovery trajectory through the *main study* using:
    I. Demographics, and comorbid physical, neuropsychological and socioeconomic factors
    II. Structural and functional MRI brain imaging, to assess metrics of cerebrovascular disease load, stroke lesion topology and brain network dynamics.

For peer review only

III. Blood biomarkers of Alzheimer's disease (phosphorylated tau forms and amyloid β42/40 ratio), neuroaxonal injury (neurofilament light, NFL and brain-derived tau), and astrocytic activation (glial fibrillary acidic protein, GFAP). The study will characterise the time course of these biomarkers over the first year after stroke and relate these to MRI measures of axonal injury and brain atrophy, as well as cognitive and functional outcomes.

IC3 Assessment Design and Development

Cognitive tests. The IC3 assessment covers 22 short tasks, spanning a wide range of cognitive domains (Table 1) followed by several clinically-validated questionnaires, designed to be completed in under 60-70 minutes. IC3 has in-built automated break reminders every 20 minutes with the additional option to take an unscheduled break at any time. IC3 is available via a web-browser on any modern device (smartphone, tablet, computer/laptop) by clicking on a link created by the study team. The IC3 test is implemented via Cognitron, a state-of-the art platform for remote neuropsychological testing rapidly being adopted by large scale population studies both in the UK and internationally [10].

Cognitive Domain			Task Name
**Primary**	**Secondary**	**Tertiary**	
Attention	Spatial ability	Space	Pear Cancellation (Visual-

For peer review only - http://bmjopen.bmj.com/site/about/guidelines.xhtml

		Object	spatial ability)
	Auditory sustained attention		Auditory Attention Task
	Visual sustained attention	Simple upper-limb movement	Simple Reaction Time
Executive	Task switching and attention		Non-Verbal Trail Making (Task Switching)
	Reasoning	Mental shifting and flexibility	Rule Finding
			Odd One Out (Pattern Recognition)
		Spatial planning	Blocks
Language	Speech production	Phonology and semantics	Naming
		Phonology	Repetition
		Phonology, fluency, and semantics	Spoken Picture Description
		Reading	Reading
	Semantics	Verbal semantics	Semantic Judgment
	Comprehension (word-picture or word-sentence matching)	Auditory and Orthographic processing	Language comprehension
Memory	Long Term		Orientation
	Short Term	Verbal working memory	Digit span
		Visuo-spatial working memory	Spatial span
		Visuo-spatial memory	Paired Associates Learning
	Episodic	Visuo-spatial	Task Recall
		Verbal	Target Word Recall from Auditory Attention Test
Number Skills			Graded Calculation
Praxis	Ideomotor action semantics		Gesture Recognition
Motor ability	Left-right upper-limb movement	Attention	Choice Reaction Time (click on left or right side of the screen)
	Multi-directional upper-limb movement		Motor Control (click on moving target)

Table 1 – Tasks tested in the IC3 and their associated cognitive domain.

For peer review only

The digital nature of the IC3 affords scalability in cognitive monitoring by being usable in both the clinical setting and home environment in the absence of a trained clinician. As well as reducing healthcare costs, this feature promotes accessibility of the assessment for physically disabled patients in whom attendance to healthcare or research setting is difficult. Compared to pen-and-paper tests, the IC3 test administration is standardised, more detailed response metrics per individual tests are provided (e.g. accuracy, reaction time and trial-by-trial variability), and real-time patient scores are calculated automatically against a large age-, education- and sex- matched control sample. The IC3 specifications are summarised in Figure 1.

Demographic and neuropsychiatric questionnaires. A number of health questionnaires and modified versions of relevant clinically-validated questionnaires are completed by the patient or carers, including Apathy Evaluation Scale [6], Fatigue Severity Scale [11], Geriatric Depression Scale [12], Instrumental Activities of Daily Living (See Table 2). Upon completion of the cognitive tests, the participants also fill in modified versions of relevant clinically-validated questionnaires, including Modified short version of Apathy Evaluation Scale [6], Modified short version of Fatigue Severity Scale [11], Short form of the Geriatric Depression Scale [12], Instrumental Activities of Daily Living [13].

Table 2

Questionnaire items included in the IC3

	**Range/Categories**

For peer review only - http://bmjopen.bmj.com/site/about/guidelines.xhtml

Age	0-100
Biological sex	Male/Female
Years of education	Forced choice answers scaled to 0-21
Handedness	Likert scale ranging from 1 to 5, where 1 is right-handed, 3 is ambidextrous and 5 is left-handed
Hand used for making responses	Left/Right
Hand impairments	Yes/No
English proficiency	Native, Excellent but non-native, moderate level, low-level
Ethnicity	White, Asian, Black, Arab, Mixed, Other
Occupational status	Unemployed, long-term sick, retired, student, self-employed, employed
Annual income	£0 - £100,000+
Existence of stroke risk factors	Diabetes, high-blood pressure, high cholesterol, heart disease, kidney disease, alcohol dependency, over-weight, smoker, ex-smoker
Existence of Medical Conditions- (followed by more probing questions if an option is selected)	Stroke, other neurological problems, depression, anxiety
If participant indicates a history of stroke	
How long ago was the stroke?	Date/Time
How many strokes	0-10
Type of stroke	Ischemic, Haemorrhagic, TIA, Subarachnoid Haemorrhage, I don't know
Problems caused by the stroke	Language, Memory, Concentration, Movement of the right arm/leg, Movement of the left arm/leg, Vision, other
Movement	Difficulties standing, sitting, and walking

At the end of the assessment the participant will be given a graphical, easy-to-understand diagram that highlights their performance against normative data (Figure 2). The normative data will be based on single time-point data collected remotely from

For peer review only

5,000 healthy controls. For tasks with normally distributed data, mild, moderate and severe impairment will be classed using suggested cut-offs of -1.5, -2.0 and -2.5 SD below the mean respectively. For tasks with ceiling effects, 90th, 95th and 99th percentiles are the suggested cut-offs.

Main IC3 study design

This prospective observational longitudinal study aims to recruit 300 patients with acute stroke and obtain repeated measures of cognition and psychosocial status at baseline, 3-months, 6-months and 12-months post ictus. IC3 is currently a single-site study at Imperial College Healthcare NHS Trust, England, and commenced recruitment in February 2022. In addition to the cognitive monitoring, all patients will be invited to take part in MRI brain imaging and blood biomarker sub-studies (Figure 3; see sub-studies for more details).

IC3 validation sub-study. A minimum of 100 patients will undergo clinically validated pen and paper cognitive screens used routinely in clinical practice including MOCA [14], star cancellation task [15] and/or the OCS. Sensitivity, specificity analyses, as well as convergence and divergence analyses will be performed. Test-retest reliability analyses will estimate the impact of learning effects on performance, and compare remote administration with in-person delivery of the assessment.

For peer review only

MRI sub-study. Clinically acquired brain imaging at the time of the acute stroke (including FLAIR and DWI sequences) will be obtained. All 300 patients will be invited to undergo additional brain MRI imaging at 3 and 12 months using a Siemens Verio 3T scanner at the Imperial College Clinical Imaging Facility. MRI measures will be used as prognostic indicators for cognitive trajectory. MRI data will be collected from additional 50 age-matched control participants for comparison. The following sequences will be acquired at each session over 60-90 minutes (breaks included):

I. Structural MRI: T1 (1 mm^3 resolution for volumetric analyses of lesion volume and brain atrophy); Susceptibility Weighted Imaging (0.8×0.8×3.0 mm^3 for identification of microbleeds); T2 FLAIR (1 mm^3 for detecting white matter hyperintensity volume (WMHV)); and NODDI (an advanced multi-shell diffusion MRI with 90 directions and 9 b_0 images) to assess axonal integrity and tissue microstructure. Lesions will be defined using established standards [16].

II. Functional MRI: Resting-state and breath-hold paradigm will measure dynamic regional brain activity and vascular reactivity respectively [17].

Blood biomarkers sub-study. Blood samples will be obtained at baseline (0-15 days post-stroke), and at 3, 6 and 12 months from 200 patients. Two EDTA (plasma) and two SST (serum) samples will be collected. Sample processing involves centrifugation at 2000g for 10 minutes at 4°C, followed by secure storage at −80°C. Additional single PAXgene sample will be collected at one time point for DNA storage for future analysis of apolipoprotein E (*APOE*) genotype and polygenic risk score measures. Patient

For peer review only

identity is kept confidential. The primary plasma biomarker of interest will be NFL [18]. Additional markers including GFAP (astrocytic activation [19]), brain-derived tau (a novel marker for neuronal injury [20]), phosphorylated tau forms and Aβ42/40 ratio (both biomarkers for Alzheimer's pathologies[21]) will be quantified. Testing will be performed at University College London via a Quanterix Simoa analyser to provide ultrasensitive measurement of concentrations.

Patient and public involvement statement

The IC3 assessment was developed and refined during a 6-months iterative process of design modifications based on patient and user feedback. We conducted feedback sessions with stroke survivors part-way through the development of our assessment to ensure that (1) our cognitive assessments are intuitive, (2) the instructions are comprehensible, (3) patients can complete the assessment with minimum supervision, and (4) the burden of assessments are acceptable. Based on these sessions, we made several improvements to the design of the assessment, primarily focusing on improving the user interface, user experience and the instructions. We also involved multiple age-matched members of the public, without a diagnosis of stroke, to ensure that our design changes were effective and to identify any issues with performing the assessment remotely. Finally, we disseminated the study design through local public outreach events (e.g., educational events for older adults, health advocacy charities) and incorporated the received feedback in the end product.

For peer review only

Study Population

Participant identification, recruitment. Supplementary Table 1 outlines the patient inclusion and exclusion criteria. Patients will be recruited from the Imperial College Healthcare NHS Trust by study personnel and the clinical team. Our goal is to recruit patients as early after stroke as is practical, and ideally within fifteen days to facilitate acute blood biomarker assessment. Control participants will be aged > 18 and without history of neurological or severe psychiatric illness.

Outcome measures

The primary outcome measure for cognition will be the IC3-derived accuracy measures for each primary cognitive domain listed in Table 1 at 1 year post ictus. Secondary outcome measures will be (a) MOCA score [14], (b) functional measures including NIH Stroke Scale [22], Modified Rankin Scale [23], Instrumental Activities of Daily Living [13] and (c) psychological measures via the Geriatric Depression Scale [12] and Apathy Evaluation Scale [6].

Data analysis plan

Cognition. Dimensionality reduction techniques, such as Principal Component Analysis, graph-based methods and hierarchical clustering will examine the inter-relationship between cognitive domains and will be used to derive latent variables. These will be used to relate to blood and imaging biomarkers. Such cognitive profiling

For peer review only

will also form the basis for future real-time optimization of the IC3 task presentation based on patients' performance.

To assess the trajectories of functional, clinical, and psychological outcome measures, we will employ linear mixed effect models. These models will include cognitive measures as the main predictors, together with standard covariates such as age, sex, lesion, and initial cognitive deficit.

Blood biomarkers. NFL, GFAP, amyloid, and tau biomarkers will be analysed to describe their dynamics after acute stroke and to compare differences between groups. Additionally, longitudinal changes in these biomarkers will be assessed within patients using non-parametric tests, where appropriate. The relationship between blood biomarker levels and continuous outcome measures at 1 year (e.g., cognitive scores), will be investigated using mixed-effects linear regression. For binarized outcome measures (e.g., favourable or unfavourable MRS scores), logistic regression will be employed.

Brain imaging. The following analyses will be performed to derive imaging predictors of cognition and relate to blood biomarker levels: i) WMHV evaluated using FSL BIANCA tool (https://fsl.fmrib.ox.ac.uk/fsl/fslwiki/BIANCA); ii) stroke lesion topology manually delineated on FLAIR images; iii) NODDI measures of axonal injury and tissue microstructure (FA, neural density and orientation dispersion index) analysed in

For peer review only

NODDI MATLAB Toolbox; iv) Cortical thickness measured using Freesurfer; v) vascular reactivity analysis [17]; vi) Resting-state fMRI analyses of the effect of stroke on functional brain networks. These measures will be compared between groups. Longitudinally, each patient will act as their own 'control' in linear regressions accounting for age, sex, initial stroke severity, lesion volume, and time. Finally, structural equation modelling will be used to determine the relationship between demographics, comorbidities, blood and brain imaging biomarkers and cognitive and functional outcome measures.

Sample Size

The primary blood biomarker predictor is NFL. Based on preliminary reported group differences [18], we expect 140 patients at baseline, and 60 patients at 3-months post stroke (when peak NFL is expected to occur), will be able to detect group differences in NFL levels between patients and controls (Wilcoxon Mann Whitney test, Cohens D=0.38 and 0.58 respectively, alpha=0.05, power=0.8, 1-tailed, allocation ratio 2:1). A much smaller sample size of 56 will be enough to detect correlations between NFL and time of sampling of the acute blood: (r^2=0.10 [18], alpha=0.05, power=0.8, 1-tailed).

The primary imaging outcome measure is WMHV. A sample size of 80 will be sufficiently powered to detect correlation between NFL and WMHV based on preliminary findings [24] (r=0.32, alpha 0.05, power 0.95). A sample size of N=70 will be sufficiently powered to detect relationship between peak plasma NFL levels and

For peer review only

binary functional outcomes (based on similar effects observed after acute brain injury (R^2 = 0.28 [25], α=0.05, power=0.9). Expecting a 20-25% loss to follow-up rate (due to patient factors and technical factors), the stated sample size of 200 in blood biomarker sub-study is sufficiently powered to detect all the above-mentioned effects.

A secondary blood biomarker predictor is raised phosphorylated tau. Based on the mean and standard deviations reported for longitudinal change in phosphorylated tau (N=374) in population of elderly cognitively normal population [26], a sample size of N=65, will have 95% power in detecting any true change in mean over 1-year period (α=0.05, effect size 0.41). Thus, the stated sample size is sufficiently powered to detect longitudinal change in phosphorylated tau.

Discussion

Cognitive impairments are highly prevalent after stroke, having detrimental implications to recovery [1]. Identifying, monitoring and managing these deficits is consistently voted the highest priority by stakeholders [5]. Given the heterogeneity of stroke and its co-morbid conditions (such as aging, neurodegeneration and background small vessel disease), there is a need for large-scale longitudinal studies that enable accurate predictions of recovery and pave the way for personalised therapy allocation. Previous longitudinal studies of post-stroke outcomes have failed to identify reliable biomarkers of recovery [7]. On the one hand, this is due to a narrow

For peer review only

focus on only limited contributors to recovery (e.g. lesion topology), despite the fact that stroke recovery is known to be inherently multi-modal [8]. On the other hand, this is due to using brief, shallow cognitive assessments in small-sized samples. Our main longitudinal study addresses this gap by coupling emerging blood and brain imaging biomarkers with a novel scalable digital technology (IC3) that allows in-depth remote monitoring of cognition, thus providing a multi-modal account of recovery.

Moreover, current stand-alone cognitive screening tools commonly used in routine clinical care are either not stroke-specific, or not comprehensive. Commonly used tools, such as MOCA [14], MMSE [27], and ACE-R [28] are tailored to detect deficits in neurodegenerative dementias and are not suitable for patients with stroke, who often have domain-specific as well as domain-general deficits [9]. Additionally, increasingly used cognitive screens designed specifically for stroke, such as the OCS, OCS Plus and CASP are not in-depth enough to allow a deep cognitive phenotyping and likely to miss the milder end of the severity spectrum. Assessments that are both stroke-specific and comprehensive, such as BCOS, are too resource-intensive for routine clinical practice or longitudinal research studies into post-stroke cognition. The IC3 assessment tool developed and applied in this study addresses these shortcomings, building the foundation for routine detailed monitoring of cognition and for scalable large-scale population-based studies of post-stroke cognitive impairment.

The blood biomarker sub-studies are expected to yield novel findings concerning the role of emerging biomarkers in neurodegenerative dementias, specifically amyloid and

For peer review only

tau entities, as well as the role of biomarkers of neuroaxonal injury, such as NFL and GFAP, in determining cognitive outcomes post-stroke. The study will investigate the longitudinal dynamics of these biomarkers in the post-acute stroke period. The integration of both brain imaging biomarkers (e.g., white matter integrity, tissue microstructure, vascular reactivity, small vessel disease load) and blood biomarkers is expected to result in a cutting-edge and easily applicable predictive models for post-stroke cognition. The IC3 study is expected to make a significant contribution to a growing number of well-designed biomarker studies aimed at post-stroke cognition (such as R4VAD [29] and Discovery [30]) which have the potential to significantly advance the treatment of a disease that affects millions of people globally every year. These studies will enable more precise targeting of cognitive rehabilitation to individuals who are most at risk of progressive decline and those who have the highest potential for recovery.

Contributions

FG designed the study and obtained funding. DCG, FG, AH, HZ, WT, PH, SB and JK contributed to the data collection plan. FG, DCG, AH, WT and PH developed IC3 platform. DCG wrote the first draft of the manuscript. All authors reviewed and approved the manuscript.

For peer review only

Conflicts of interest

PH is the co-founder and chief executive of H2 Cognitive Designs LTD, for which he receives remuneration. HZ has served at scientific advisory boards and/or as a consultant for a number of commercial companies and is a co-founder of Brain Biomarker Solutions.

For peer review only

Data management and regulatory approval

All data collected will be pseudo-anonymised and securely stored on university server. Blood samples will be stored at the Imperial College Clinical Research Facility and Imperial College Healthcare NHS Trust. Data management procedures will be compliant with both Imperial College London guidelines and GDPR regulations.

Funding

Study is funded by the Medical Research Council (MR/T001402/1). The clinical study is registered under NCT05885295.

Informed Consent and Ethical Approval

This study has been approval by South West - Frenchay Research Ethics Committee (IRAS 299333), and authorized by the UK's Health Research Authority. All participants satisfying the inclusion and exclusion criteria will be approached and consented. If a patient is unable to provide fully informed consent, for example due to severe aphasia, we will assent with permission from a consultee. Patients that have been assented will be reconsented if they are subsequently able to provide full informed consent. The consent procedure will be carried out in strict compliance with national legislation and General Data Protection Regulation.

References

[1] Douiri A, Rudd AG, Wolfe CD. Prevalence of poststroke cognitive impairment: South London stroke register 1995–2010. *Stroke* 2013; 44: 138–145.

[2] Grönberg A, Henriksson I, Stenman M, et al. Incidence of Aphasia in Ischemic Stroke. *Neuroepidemiology* 2022; 56: 174–182.

[3] Obaid M, Flach C, Marshall I, et al. Long-Term Outcomes in Stroke Patients with Cognitive Impairment: A Population-Based Study. *Geriatrics* 2020; 5: 32.

[4] Hill G, Regan S, Francis R, et al. Research priorities to improve stroke outcomes. *Lancet Neurol* 2022; 21: 312–313.

[5] Pollock A, St George B, Fenton M, et al. Top 10 Research Priorities Relating to Life after Stroke – Consensus from Stroke Survivors, Caregivers, and Health Professionals. *Int J Stroke* 2014; 9: 313–320.

[6] Quinn TJ, Elliott E, Langhorne P. Cognitive and Mood Assessment Tools for Use in Stroke. *Stroke* 2018; 49: 483–490.

For peer review only

[7] Boyd LA, Hayward KS, Ward NS, et al. Biomarkers of stroke recovery: Consensus-based core recommendations from the Stroke Recovery and Rehabilitation Roundtable. *Int J Stroke* 2017; 12: 480–493.

[8] Simpkins AN, Janowski M, Oz HS, et al. Biomarker Application for Precision Medicine in Stroke. *Transl Stroke Res* 2020; 11: 615–627.

[9] Demeyere N, Riddoch MJ, Slavkova ED, et al. Domain-specific versus generalized cognitive screening in acute stroke. *J Neurol* 2016; 263: 306–315.

[10] Hampshire A, Hellyer PJ, Soreq E, et al. Associations between dimensions of behaviour, personality traits, and mental-health during the COVID-19 pandemic in the United Kingdom. *Nat Commun* 2021; 12: 4111.

[11] Krupp LB, LaRocca NG, Muir-Nash J, et al. The fatigue severity scale: application to patients with multiple sclerosis and systemic lupus erythematosus. *Arch Neurol* 1989; 46: 1121–1123.

[12] Herrmann N, Mittmann N, Silver IL, et al. A validation study of The Geriatric Depression Scale short form. *Int J Geriatr Psychiatry* 1996; 11: 457–460.

[13] Lawton MP, Brody EM. Assessment of older people: self-maintaining and instrumental activities of daily living. *The gerontologist* 1969; 9: 179–186.

[14] Nasreddine ZS, Phillips NA, Bédirian V, et al. The Montreal Cognitive Assessment, MoCA: A Brief Screening Tool For Mild Cognitive Impairment. *J Am Geriatr Soc* 2005; 53: 695–699.

[15] Friedman PJ. The Star Cancellation Test in acute stroke. *Clin Rehabil* 1992; 6: 23–30.

[16] Wardlaw JM, Smith EE, Biessels GJ, et al. Neuroimaging standards for research into small vessel disease and its contribution to ageing and neurodegeneration. *Lancet Neurol* 2013; 12: 822–838.

[17] Geranmayeh F, Wise RJS, Leech R, et al. Measuring vascular reactivity with breath-holds after stroke: A method to aid interpretation of group-level BOLD

For peer review only

signal changes in longitudinal fMRI studies. *Hum Brain Mapp* 2015; 36: 1755–1771.

[18] Pedersen A, Stanne TM, Nilsson S, et al. Circulating neurofilament light in ischemic stroke: temporal profile and outcome prediction. *J Neurol* 2019; 266: 2796–2806.

[19] Chatterjee P, Doré V, Pedrini S, et al. Plasma Glial Fibrillary Acidic Protein Is Associated with 18F-SMBT-1 PET: Two Putative Astrocyte Reactivity Biomarkers for Alzheimer's Disease. *J Alzheimers Dis JAD* 2023; 92: 615–628.

[20] Gonzalez-Ortiz F, Turton M, Kac PR, et al. Brain-derived tau: a novel blood-based biomarker for Alzheimer's disease-type neurodegeneration. *Brain J Neurol* 2023; 146: 1152–1165.

[21] Zetterberg H, Bendlin BB. Biomarkers for Alzheimer's disease-preparing for a new era of disease-modifying therapies. *Mol Psychiatry* 2021; 26: 296–308.

[22] Wityk RJ, Pessin MS, Kaplan RF, et al. Serial assessment of acute stroke using the NIH Stroke Scale. *Stroke* 1994; 25: 362–365.

[23] van Swieten JC, Koudstaal PJ, Visser MC, et al. Interobserver agreement for the assessment of handicap in stroke patients. *Stroke* 1988; 19: 604–607.

[24] Gattringer T, Pinter D, Enzinger C, et al. Serum neurofilament light is sensitive to active cerebral small vessel disease. *Neurology* 2017; 89: 2108–2114.

[25] Graham NSN, Zimmerman KA, Moro F, et al. Axonal marker neurofilament light predicts long-term outcomes and progressive neurodegeneration after traumatic brain injury. *Sci Transl Med* 2021; 13: eabg9922.

[26] Moscoso A, Grothe MJ, Ashton NJ, et al. Longitudinal associations of blood phosphorylated Tau181 and neurofilament light chain with neurodegeneration in Alzheimer disease. *JAMA Neurol* 2021; 78: 396–406.

[27] Kurlowicz L, Wallace M. The mini-mental state examination (MMSE). *Journal of gerontological nursing* 1999; 25: 8–9.

For peer review only

[28] Mioshi E, Dawson K, Mitchell J, et al. The Addenbrooke's Cognitive Examination Revised (ACE-R): a brief cognitive test battery for dementia screening. *Int J Geriatr Psychiatry* 2006; 21: 1078–1085.

[29] Wardlaw JM, Doubal F, Brown R, et al. Rates, risks and routes to reduce vascular dementia (R4vad), a UK-wide multicentre prospective observational cohort study of cognition after stroke: Protocol. *Eur Stroke J* 2021; 6: 89–101.

[30] Rost NS, Meschia JF, Gottesman R, et al. Cognitive Impairment and Dementia After Stroke: Design and Rationale for the DISCOVERY Study. *Stroke*; 52. Epub ahead of print August 2021. DOI: 10.1161/STROKEAHA.120.031611.

For peer review only

Supplementary Table 1 - Inclusion and Exclusion Criteria for Patients

Inclusion Criteria	***Exclusion Criteria***
Aged > 18	*For the main study:*
Evidence of confirmed stroke	Pre-stroke diagnosis of dementia<br>Inability to follow instructions
Ability to concentrate for 15 minutes at a time to engage with cognitive testing	Severe visuo-spatial problems, fatigue, or mental health problems
	Severe hearing impairment in the presence of reading comprehension impairment
	For the imaging sub-study:
	Pregnancy
	Ferromagnetic implants
	Claustrophobia

For peer review only

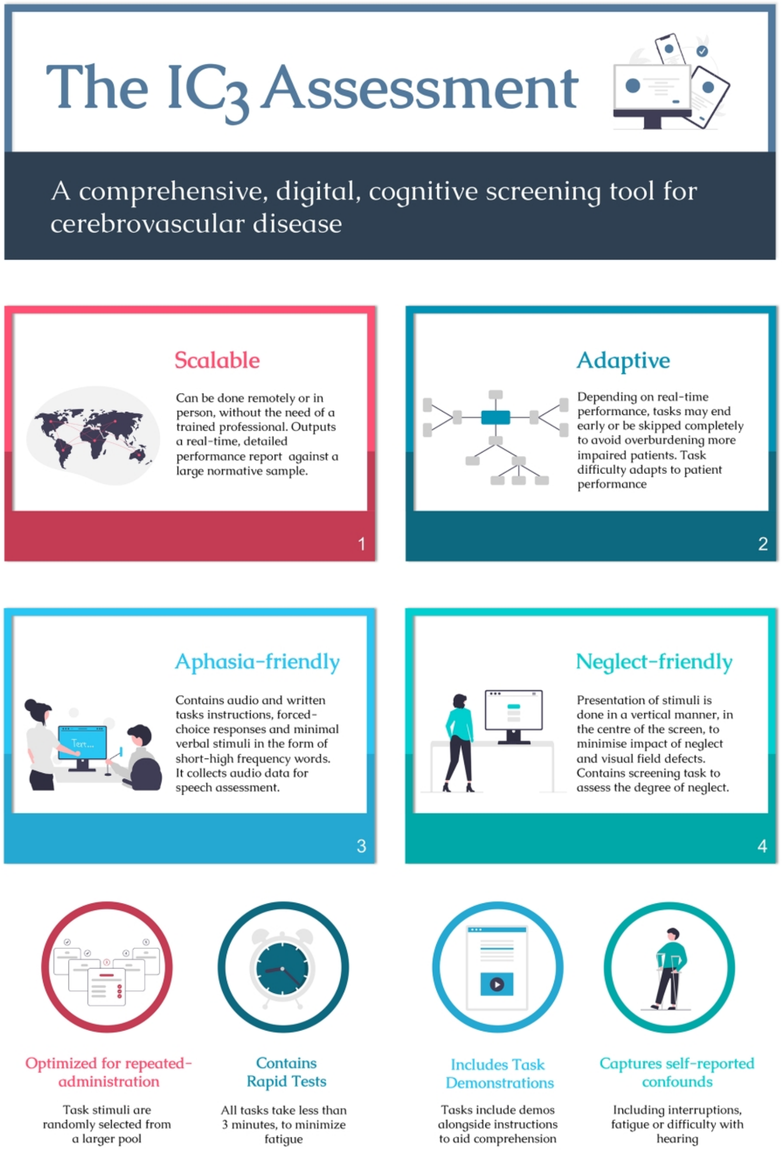

158x228mm (300 x 300 DPI)

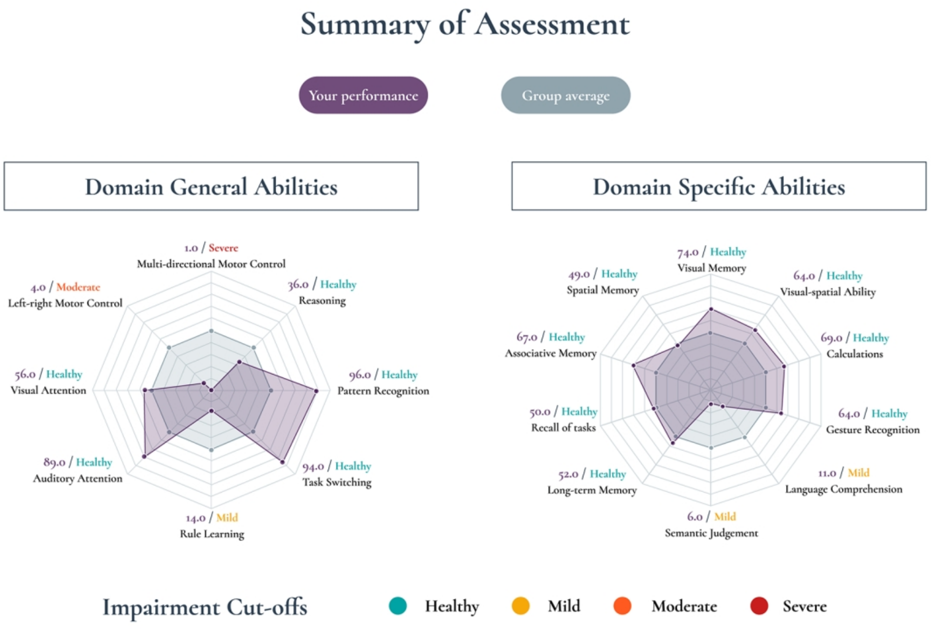

150x106mm (300 x 300 DPI)

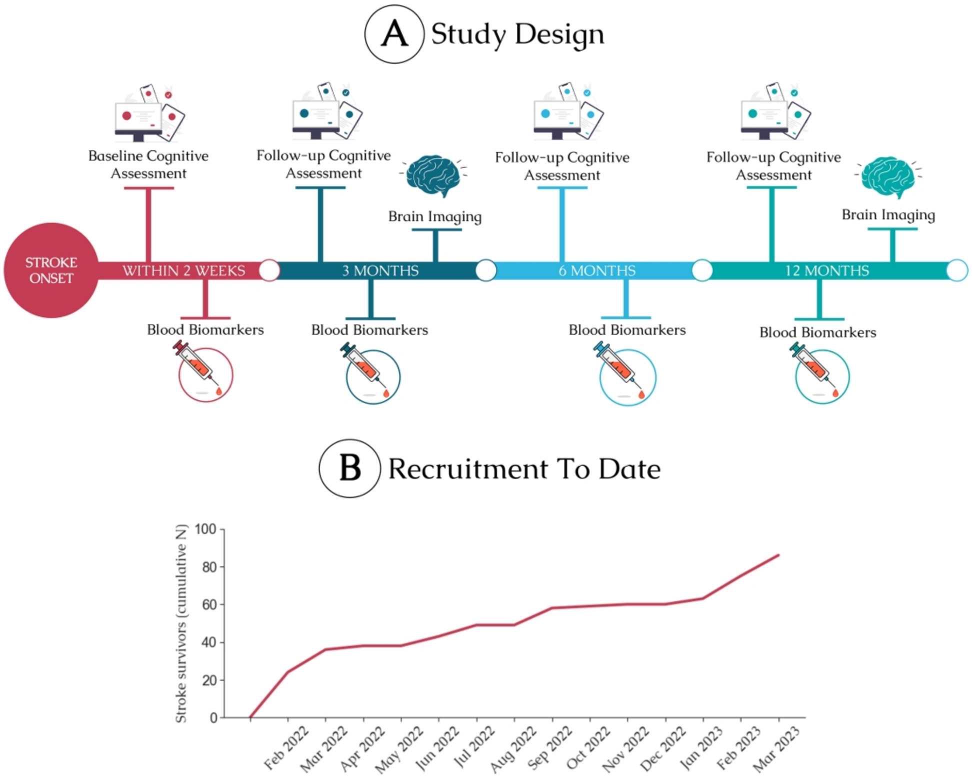

188x156mm (300 x 300 DPI)

Reporting checklist for cross sectional study.

Based on the STROBE cross sectional guidelines.

Instructions to authors

Complete this checklist by entering the page numbers from your manuscript where readers will find each of the items listed below.

Your article may not currently address all the items on the checklist. Please modify your text to include the missing information. If you are certain that an item does not apply, please write "n/a" and provide a short explanation.

Upload your completed checklist as an extra file when you submit to a journal.

In your methods section, say that you used the STROBE cross sectionalreporting guidelines, and cite them as:

von Elm E, Altman DG, Egger M, Pocock SJ, Gotzsche PC, Vandenbroucke JP. The Strengthening the Reporting of Observational Studies in Epidemiology (STROBE) Statement: guidelines for reporting observational studies.

		Reporting Item	Page Number
**Title and abstract**			
Title	#1a	Indicate the study's design with a commonly used term in the title or the abstract	1
Abstract	#1b	Provide in the abstract an informative and balanced summary of what was done and what was found	1
**Introduction**			
Background / rationale	#2	Explain the scientific background and rationale for the investigation being reported	1
Objectives	#3	State specific objectives, including any prespecified hypotheses	4
**Methods**			
Study design	#4	Present key elements of study design early in the paper	8-9
Setting	#5	Describe the setting, locations, and relevant dates, including periods of	9-10

For peer review only

		recruitment, exposure, follow-up, and data collection	
Eligibility criteria	#6a	Give the eligibility criteria, and the sources and methods of selection of participants.	9
	#7	Clearly define all outcomes, exposures, predictors, potential confounders, and effect modifiers. Give diagnostic criteria, if applicable	10
Data sources / measurement	#8	For each variable of interest give sources of data and details of methods of assessment (measurement). Describe comparability of assessment methods if there is more than one group. Give information separately for for exposed and unexposed groups if applicable.	10-11
Bias	#9	Describe any efforts to address potential sources of bias	11-12
Study size	#10	Explain how the study size was arrived at	11-12
Quantitative variables	#11	Explain how quantitative variables were handled in the analyses. If applicable, describe which groupings were chosen, and why	10-11
Statistical methods	#12a	Describe all statistical methods, including those used to control for confounding	10-11
Statistical methods	#12b	Describe any methods used to examine subgroups and interactions	10-11
Statistical methods	#12c	Explain how missing data were addressed	n/a
Statistical methods	#12d	If applicable, describe analytical methods taking account of sampling strategy	n/a
Statistical methods	#12e	Describe any sensitivity analyses	8
**Results**			
Participants	#13a	Report numbers of individuals at each stage of study—eg numbers potentially eligible, examined for eligibility, confirmed eligible, included in the study, completing follow-up, and analysed. Give information separately for for exposed and unexposed groups if applicable.	n/a
Participants	#13b	Give reasons for non-participation at each stage	n/a
Participants	#13c	Consider use of a flow diagram	n/a

For peer review only

Descriptive data	#14a	Give characteristics of study participants (eg demographic, clinical, social) and information on exposures and potential confounders. Give information separately for exposed and unexposed groups if applicable.	n/a
Descriptive data	#14b	Indicate number of participants with missing data for each variable of interest	n/a
Outcome data	#15	Report numbers of outcome events or summary measures. Give information separately for exposed and unexposed groups if applicable.	n/a
Main results	#16a	Give unadjusted estimates and, if applicable, confounder-adjusted estimates and their precision (eg, 95% confidence interval). Make clear which confounders were adjusted for and why they were included	n/a
Main results	#16b	Report category boundaries when continuous variables were categorized	n/a
Main results	#16c	If relevant, consider translating estimates of relative risk into absolute risk for a meaningful time period	n/a
Other analyses	#17	Report other analyses done—e.g., analyses of subgroups and interactions, and sensitivity analyses	n/a
**Discussion**			
Key results	#18	Summarise key results with reference to study objectives	n/a
Limitations	#19	Discuss limitations of the study, taking into account sources of potential bias or imprecision. Discuss both direction and magnitude of any potential bias.	n/a
Interpretation	#20	Give a cautious overall interpretation considering objectives, limitations, multiplicity of analyses, results from similar studies, and other relevant evidence.	n/a
Generalisability	#21	Discuss the generalisability (external validity) of the study results	n/a
**Other Information**			
Funding	#22	Give the source of funding and the role of the funders for the present study and, if applicable, for the original study on which the present article is based	14

The STROBE checklist is distributed under the terms of the Creative Commons Attribution License CC-BY. This checklist was completed on 07. June 2023 using https://www.goodreports.org/, a tool made by the EQUATOR Network in collaboration with Penelope.ai

For peer review only

BMJ Open

IC3 Protocol – A longitudinal observational study of cognition after stroke using novel digital health technology

Journal:	*BMJ Open*
Manuscript ID	bmjopen-2023-076653.R1
Article Type:	Protocol
Date Submitted by the Author:	18-Oct-2023
Complete List of Authors:	Gruia, Dragos-Cristian; Imperial College London, Brain Sciences; Imperial College Healthcare NHS Trust<br>Trender, William; Imperial College London, Brain Sciences<br>Hellyer, Peter; King's College London, Centre for Neuroimaging Sciences, IoPPN<br>Banerjee, Soma; Imperial College Healthcare NHS Trust<br>Kwan, Joseph; Imperial College Healthcare NHS Trust<br>Zetterberg, Henrik; UCL, Department of Neurodegenerative Disease, Institute of Neurology; University of Gothenburg, Department of Psychiatry and Neurochemistry<br>Hampshire, Adam; Imperial College London, Brain Sciences<br>Geranmayeh, Fatemeh; Imperial College Healthcare NHS Trust; Imperial College London, Brain Sciences
**Primary Subject Heading**:	Neurology
Secondary Subject Heading:	Geriatric medicine, Neurology
Keywords:	Stroke < NEUROLOGY, Stroke medicine < INTERNAL MEDICINE, Magnetic resonance imaging < RADIOLOGY & IMAGING

SCHOLARONE™
Manuscripts

IC3 Protocol – A longitudinal observational study of cognition after stroke using novel digital health technology

Dragos C. Gruia[1,3], William Trender[1], Peter Hellyer[2], Soma Banerjee[1,3], Joseph Kwan[1,3], Henrik Zetterberg[4-9], Adam Hampshire[1], Fatemeh Geranmayeh[1,3]

1- Department of Brain Sciences, Imperial College London, London, W12 0NN, UK.
2- Centre for Neuroimaging Sciences, IoPPN, King's College London. SE5 8AF, London, UK.
3- Imperial College NHS Healthcare Trust, London W6 8RF, UK.
4- Department of Psychiatry and Neurochemistry, Institute of Neuroscience and Physiology, the Sahlgrenska Academy at the University of Gothenburg, Mölndal, Sweden
5- Department of Neurodegenerative Disease, UCL Institute of Neurology, Queen Square, London, UK

For peer review only - http://bmjopen.bmj.com/site/about/guidelines.xhtml

6- Clinical Neurochemistry Laboratory, Sahlgrenska University Hospital, Mölndal, Sweden

7- UK Dementia Research Institute at UCL, London, UK

8- Hong Kong Center for Neurodegenerative Diseases, Clear Water Bay, Hong Kong, China

9- Wisconsin Alzheimer's Disease Research Center, University of Wisconsin School of Medicine and Public Health, University of Wisconsin-Madison, Madison, WI, USA

Corresponding author:

Dr Fatemeh Geranmayeh

Clinical Language and Cognition group. Department of Brain Sciences. Imperial College London. London W12 0NN. UK

Email: fatemeh.geranmayeh00@imperial.ac.uk

Keywords:

Cognitive testing, stroke, cerebrovascular disease, observational study, biomarkers

Abstract

For peer review only - http://bmjopen.bmj.com/site/about/guidelines.xhtml

Introduction. Stroke is a major cause of death and disability worldwide, frequently resulting in persistent cognitive deficits among survivors. These deficits negatively impact recovery and therapy engagement, and their treatment is consistently rated as high priority by stakeholders and clinicians. Although clinical guidelines endorse cognitive screening for post-stroke management, there is currently no gold standard approach for identifying cognitive deficits after stroke, and clinical stroke services lack the capacity for long-term cognitive monitoring and care. Currently available assessment tools are either not stroke-specific, not in-depth or lack scalability, leading to heterogeneity in patient assessments. **Methods and Analysis.** To address these challenges, a cost-effective, scalable, and comprehensive screening tool is needed to provide a stroke-specific assessment of cognition. The current study presents such a novel digital tool, the Imperial Comprehensive Cognitive Assessment in Cerebrovascular Disease (IC3), designed to detect both domain-general and domain-specific cognitive deficits in patients after stroke with minimal input from a health professional. To ensure its reliability, we will utilise multiple validation approaches, and aim to recruit a large normative sample of age-, gender-, and education-matched UK-based controls. Moreover, the IC3 assessment will be integrated within a larger prospective observational longitudinal clinical trial, where post-stroke cognition will be examined in tandem with brain imaging and blood biomarkers to identify novel multimodal biomarkers of recovery after stroke. This study will enable deeper cognitive

For peer review only - http://bmjopen.bmj.com/site/about/guidelines.xhtml

phenotyping of patients at a large scale, whilst identifying those with highest risk of progressive cognitive decline, as well as those with greatest potential for recovery. **Ethics and dissemination.** This study has been approval by South West - Frenchay Research Ethics Committee (IRAS 299333), and authorized by the UK's Health Research Authority. Results from the study will be disseminated at conferences and within peer-reviewed journals. **Study registration.** The study is registered as an observational trial under NCT05885295.

Strengths and limitations

- A major methodological strength of the study involves the development of the first digital screening technology that allows remote monitoring of stroke-specific cognitive deficits in a manner that is cost-effective and suitable for large-scale population-based investigations.

- Through its digital and conventional assessments, the current study provides a scalable yet deep cognitive phenotyping of both domain-specific and domain-general impairments, longitudinally mapping post-stroke recovery trajectory.

- A particular strength of the study is the comprehensive collection of multimodal data (behaviour, brain imaging and blood) that allows the development of novel quantifiable biomarkers of post-stroke recovery.

- A limitation of the digital assessment is that it is designed to target stroke survivors with mild to moderate cognitive impairment, thus likely will have limited applicability in patients with severe deficits.

For peer review only - http://bmjopen.bmj.com/site/about/guidelines.xhtml

- While the use of technology to administer unsupervised cognitive assessments at a large scale facilitates improved diagnostic and monitoring capabilities, it may also lead to systematic exclusion of those with limited access to technology.

For peer review only

Introduction

Stroke is a leading cause of death and disability globally, with frequent cognitive sequalae affecting three-quarters of survivors [1]. The spectrum of cognitive deficits includes background 'vascular cognitive impairment' and more domain-specific deficits, such as aphasia which affects a third of patients [2]. Collectively, these impairments negatively impact post-stroke recovery and engagement with therapy [3]. Furthermore, there is a high failure rate of post-stroke cognitive rehabilitation trials, due to our poor understanding of individual patient-level mechanisms of post-stroke cognitive recovery and the ensuing inadequate stratification of both treatment responders and non-responders in the same clinical trials. It is therefore important to understand the factors that affect cognitive recovery post-stroke, a topic increasingly recognised by funders and stakeholders as a high priority research area [4–6].

For peer review only - http://bmjopen.bmj.com/site/about/guidelines.xhtml

Although clinical guidelines have been recently updated to recommend cognitive screening for post-stroke management, there is currently no gold standard approach for identifying cognitive deficits after stroke [7]. Clinicians and researchers often select cognitive tests depending on preference, familiarity and availability, leaving little prospect for generalizability of their findings. Moreover, clinical stroke services do not have the capacity to provide long-term cognitive monitoring and care for stroke survivors who have unpredictable recovery trajectories [3, 8]. This challenge is further compounded by the lack of cost-effective cognitive tools suitable for long-term monitoring, and the reliance of such monitoring on resource-stretched healthcare professionals.

Availability of a cost-effective, reliable, and comprehensive screening tool that provides a stroke-specific deep phenotyping of cognition would be a game-changer. In the clinical setting, it would improve the detection rate and monitoring of cognitive deficits post stroke, and in the research setting it would facilitate much needed large-scale population-based mechanistic studies aimed at understanding the mechanisms of cognitive recovery. Here, we present such novel digital adaptive tool: The Imperial Comprehensive Cognitive Assessment in Cerebrovascular Disease (IC3). IC3 is a digital assessment designed to require minimal input from a clinician in detecting both domain-general and domain-specific cognitive deficits in patients after stroke. To ensure its reliability, several validation studies will be conducted against established clinical screening tools, and normative samples will be computed using representative age-, gender- and education-matched UK-based controls.

For peer review only

For peer review only - http://bmjopen.bmj.com/site/about/guidelines.xhtml

In line with recent efforts in identifying biomarkers of post-stroke recovery [9], the IC3 tool will be integrated within a larger prospective longitudinal clinical study, where post-stroke cognition will be examined in tandem with brain imaging and novel blood biomarkers. This study is entitled "Understanding factors affecting cognitive function in cerebrovascular disease" and will hereafter be referred to as the '*main study*'.

For peer review only

Methods and Analysis

Aims

1. Develop an adaptive, scalable, self-administered, digital comprehensive cognitive screening tool to detect cognitive impairments in patients with cerebrovascular disease. The IC3 will:
    I. Detect both domain-general (e.g. executive function) and domain-specific (e.g. aphasia, apraxia, neglect) deficits post-stroke [10].
    II. Allow monitoring to occur at scale, in a cost-efficient manner, as test administration can occur independent of trained professionals.
    III. Minimise the effects of neglect or aphasia on cognitive assessments.
    IV. Have high test-re-test reliability for repeated-testing longitudinally.
2. Validate the IC3 against commonly used cognitive screening tools.

For peer review only - http://bmjopen.bmj.com/site/about/guidelines.xhtml

3. Use IC3 in a cohort of stroke survivors to map the cognitive recovery trajectory over the course of the first year after stroke.
4. Identify novel biomarkers of cognitive outcome (at 1 year) and recovery trajectory through the *main study* using:
   I. Demographics, and comorbid physical, neuropsychological and socioeconomic factors
   II. Structural and functional MRI brain imaging, to assess metrics of cerebrovascular disease load, stroke lesion topology and brain network dynamics.
   III. Blood biomarkers of Alzheimer's disease (phosphorylated tau forms and amyloid β42/40 ratio), neuroaxonal injury (neurofilament light, NFL and brain-derived tau), and astrocytic activation (glial fibrillary acidic protein, GFAP). The study will characterise the time course of these biomarkers over the first year after stroke and relate these to MRI measures of axonal injury and brain atrophy, as well as cognitive and functional outcomes.

IC3 Assessment Design and Development

Cognitive tests. The IC3 assessment covers 22 short tasks, spanning a wide range of cognitive domains (Table 1) followed by several clinically-validated questionnaires, designed to be completed in under 60-70 minutes. IC3 has in-built automated break

For peer review only - http://bmjopen.bmj.com/site/about/guidelines.xhtml

reminders every 20 minutes with the additional option to take an unscheduled break at any time. IC3 is available via a web-browser on any modern device (smartphone, tablet, computer/laptop) by clicking on a link created by the study team. The IC3 test is implemented via Cognitron, a state-of-the art platform for remote neuropsychological testing rapidly being adopted by large scale population studies both in the UK and internationally [11].

For peer review only

Cognitive Domain			Task Name
**Primary**	**Secondary**	**Tertiary**	
Attention	Spatial ability	Space	Pear Cancellation (Visual-spatial ability)
		Object	
	Auditory sustained attention		Auditory Attention Task
	Visual sustained attention	Simple upper-limb movement	Simple Reaction Time
Executive	Task switching and attention		Non-Verbal Trail Making (Task Switching)
	Reasoning	Mental shifting and flexibility	Rule Finding
			Odd One Out (Pattern Recognition)
		Spatial planning	Blocks
Language	Speech production	Phonology and semantics	Naming
		Phonology	Repetition
		Phonology, fluency, and semantics	Spoken Picture Description
		Reading	Reading
	Semantics	Verbal semantics	Semantic Judgment
	Comprehension (word-picture or word-sentence matching)	Auditory and Orthographic processing	Language comprehension
Memory	Long Term		Orientation
	Short Term	Verbal working memory	Digit span
		Visuo-spatial working memory	Spatial span
		Visuo-spatial	Paired Associates Learning

For peer review only - http://bmjopen.bmj.com/site/about/guidelines.xhtml

		memory	
	Episodic	Visuo-spatial	Task Recall
		Verbal	Target Word Recall from Auditory Attention Test
Number Skills			Graded Calculation
Praxis	Ideomotor action semantics		Gesture Recognition
Motor ability	Left-right upper-limb movement	Attention	Choice Reaction Time (click on left or right side of the screen)
	Multi-directional upper-limb movement		Motor Control (click on moving target)

Table 1 – Tasks tested in the IC3 and their associated cognitive domain.

The digital nature of the IC3 affords scalability in cognitive monitoring by being usable in both the clinical setting and home environment in the absence of a trained clinician. As well as reducing healthcare costs, this feature promotes accessibility of the assessment for physically disabled patients in whom attendance to healthcare or research setting is difficult. Compared to pen-and-paper tests, the IC3 test administration is standardised, more detailed response metrics per individual tests are provided (e.g. accuracy, reaction time and trial-by-trial variability), and real-time patient scores are calculated automatically against a large age-, education- and sex- matched control sample. The IC3 specifications are summarised in Figure 1.

Demographic and neuropsychiatric questionnaires. A number of health questionnaires and modified versions of relevant clinically-validated questionnaires are completed by the patient or carers, including Apathy Evaluation Scale [7], Fatigue Severity Scale [12], Geriatric Depression Scale [13], Instrumental Activities of Daily Living (See Table

For peer review only - http://bmjopen.bmj.com/site/about/guidelines.xhtml

2). Upon completion of the cognitive tests, the participants also fill in modified versions of relevant clinically-validated questionnaires, including Modified short version of Apathy Evaluation Scale [7], Modified short version of Fatigue Severity Scale [12], Short form of the Geriatric Depression Scale [13], Instrumental Activities of Daily Living [14].

Table 2

Questionnaire items included in the IC3

	**Range/Categories**
Age	0-100
Biological sex	Male/Female
Years of education	Forced choice answers scaled to 0-21
Handedness	Likert scale ranging from 1 to 5, where 1 is right-handed, 3 is ambidextrous and 5 is left-handed
Hand used for making responses	Left/Right
Hand impairments	Yes/No
English proficiency	Native, Excellent but non-native, moderate level, low-level
Ethnicity	White, Asian, Black, Arab, Mixed, Other
Occupational status	Unemployed, long-term sick, retired, student, self-employed, employed
Annual income	£0 - £100,000+
Existence of stroke risk factors	Diabetes, high-blood pressure, high cholesterol, heart disease, kidney disease, alcohol dependency, over-weight, smoker, ex-smoker
Existence of Medical Conditions- (followed by more probing questions if an option is selected)	Stroke, other neurological problems, depression, anxiety
If participant indicates a history of stroke	

For peer review only - http://bmjopen.bmj.com/site/about/guidelines.xhtml

How long ago was the stroke?	Date/Time
How many strokes	0-10
Type of stroke	Ischemic, Haemorrhagic, TIA, Subarachnoid Haemorrhage, I don't know
Problems caused by the stroke	Language, Memory, Concentration, Movement of the right arm/leg, Movement of the left arm/leg, Vision, other
Movement	Difficulties in standing, sitting, and in sit-to-stand

For peer review only

At the end of the assessment the participant will be given a graphical, easy-to-understand diagram that highlights their performance against normative data (Figure 2). The normative data will be based on single time-point data collected remotely from 5,000 healthy controls. For tasks with normally distributed data, mild, moderate and severe impairment will be classed using suggested cut-offs of -1.5, -2.0 and -2.5 SD below the mean respectively. For tasks with ceiling effects, 90th, 95th and 99th percentiles are the suggested cut-offs.

Main IC3 study design

This prospective observational longitudinal study aims to recruit 300 patients with acute stroke and obtain repeated measures of cognition and psychosocial status at baseline, 3-months, 6-months and 12-months post ictus. IC3 is currently a single-site study at Imperial College Healthcare NHS Trust, England, and commenced recruitment in February 2022. In addition to the cognitive monitoring, all patients will be invited to take part in MRI brain imaging and blood biomarker sub-studies (Figure 3; see sub-studies for more details).

IC3 validation sub-study. A minimum of 100 patients will undergo clinically validated pen and paper cognitive screens used routinely in clinical practice including the Montreal Cognitive Assessment (MOCA) [15], star cancellation task [16] and/or the Oxford Cognitive Screen (OCS). Sensitivity, specificity analyses, as well as convergence and divergence analyses will be performed. Test-retest reliability analyses will estimate the impact of learning effects on performance, and compare remote administration with in-person delivery of the assessment.

MRI sub-study. Clinically acquired brain imaging at the time of the acute stroke (including fluid-attenuated inversion recovery (FLAIR) and diffusion-weighted imaging (DWI) sequences) will be obtained. All 300 patients will be invited to undergo additional brain MRI imaging at 3 and 12 months using a Siemens Verio 3T scanner at the Imperial College Clinical Imaging Facility. MRI measures will be used as prognostic indicators for cognitive trajectory. MRI data will be collected from additional 50 age-matched control participants for comparison. The following sequences will be acquired at each session over 60-90 minutes (breaks included):

I. Structural MRI: T1 (1 mm^3 resolution for volumetric analyses of lesion volume and brain atrophy); Susceptibility Weighted Imaging ($0.8 \times 0.8 \times 3.0$ mm^3 for identification of microbleeds); T2 fluid-attenuated inversion recovery (FLAIR, 1 mm^3 for detecting white matter hyperintensity volume (WMHV)); and neurite orientation dispersion and density imaging (NODDI, an advanced multi-shell

For peer review only - http://bmjopen.bmj.com/site/about/guidelines.xhtml

diffusion MRI with 90 directions and 9 b_0 images) to assess axonal integrity and tissue microstructure. Lesions will be defined using established standards [17].

II. Functional MRI: Resting-state and breath-hold paradigm will measure dynamic regional brain activity and vascular reactivity respectively [18].

Blood biomarkers sub-study. Blood samples will be obtained at baseline (0-15 days post-stroke), and at 3, 6 and 12 months from 200 patients. Two EDTA (plasma) and two SST (serum) samples will be collected. Sample processing involves centrifugation at 2000g for 10 minutes at 4°C, followed by secure storage at −80°C. Additional single PAXgene sample will be collected at one time point for DNA storage for future analysis of apolipoprotein E (*APOE*) genotype and polygenic risk score measures. Patient identity is kept confidential. The primary plasma biomarker of interest will be neurofilament light (NFL) [19]. Additional markers including glial fibrillary acidic protein (GFAP, for astrocytic activation [20]), brain-derived tau (a novel marker for neuronal injury [21]), phosphorylated tau forms and Aβ42/40 ratio (both biomarkers for Alzheimer's pathologies[22]) will be quantified. Testing will be performed at University College London via a Quanterix Simoa analyser to provide ultrasensitive measurement of concentrations.

Patient and public involvement statement

For peer review only - http://bmjopen.bmj.com/site/about/guidelines.xhtml

The IC3 assessment was developed and refined during a 6-months iterative process of design modifications based on patient and user feedback. We conducted feedback sessions with stroke survivors part-way through the development of our assessment to ensure that (1) our cognitive assessments are intuitive, (2) the instructions are comprehensible, (3) patients can complete the assessment with minimum supervision, and (4) the burden of assessments are acceptable. Based on these sessions, we made several improvements to the design of the assessment, primarily focusing on improving the user interface, user experience and the instructions. We also involved multiple age-matched members of the public, without a diagnosis of stroke, to ensure that our design changes were effective and to identify any issues with performing the assessment remotely. Finally, we disseminated the study design through local public outreach events (e.g., educational events for older adults, health advocacy charities) and incorporated the received feedback in the end product.

Study Population

Participant identification, recruitment. Supplementary Table 1 outlines the patient inclusion and exclusion criteria. Patients will be recruited from the Imperial College Healthcare NHS Trust by study personnel and the clinical team. Our goal is to recruit patients as early after stroke as is practical, and ideally within fifteen days to facilitate acute blood biomarker assessment. Control participants will be aged > 18 and without history of neurological or severe psychiatric illness.

For peer review only

Outcome measures

The primary outcome measure for cognition will be the IC3-derived accuracy measures for each primary cognitive domain listed in Table 1 at 1 year post ictus. Secondary outcome measures will be (a) MOCA score [15], (b) functional measures including National Institute of Health (NIH) Stroke Scale [23], Modified Rankin Scale [24], Instrumental Activities of Daily Living [14] and (c) psychological measures via the Geriatric Depression Scale [13] and Apathy Evaluation Scale [7].

Data analysis plan

Cognition. Dimensionality reduction techniques, such as Principal Component Analysis, graph-based methods and hierarchical clustering will examine the inter-relationship between cognitive domains and will be used to derive latent variables. These will be used to relate to blood and imaging biomarkers. Such cognitive profiling will also form the basis for future real-time optimization of the IC3 task presentation based on patients' performance.

To assess the trajectories of functional, clinical, and psychological outcome measures, we will employ linear mixed effect models. These models will include cognitive measures as the main predictors, together with standard covariates such as age, sex, lesion, and initial cognitive deficit.

For peer review only

Blood biomarkers. Neurofilament light (NFL), glial fibrillary acidic protein (GFAP), amyloid, and tau biomarkers will be analysed to describe their dynamics after acute stroke and to compare differences between groups. Additionally, longitudinal changes in these biomarkers will be assessed within patients using non-parametric tests, where appropriate. The relationship between blood biomarker levels and continuous outcome measures at 1 year (e.g., cognitive scores), will be investigated using mixed-effects linear regression. For binarized outcome measures (e.g., favourable or unfavourable MRS scores), logistic regression will be employed.

Brain imaging. The following analyses will be performed to derive imaging predictors of cognition and relate to blood biomarker levels: i) white matter hyperintensity volume (WMHV) evaluated using FSL Brain Intensity Abnormality Classification Algorithm (BIANCA) package (https://fsl.fmrib.ox.ac.uk/fsl/fslwiki/BIANCA); ii) stroke lesion topology manually delineated on fluid-attenuated inversion recovery (FLAIR) images; iii) neurite orientation dispersion and density imaging (NODDI) measures of axonal injury and tissue microstructure (fractional anisotropy (FA), neural density and orientation dispersion index) analysed in NODDI MATLAB Toolbox; iv) Cortical thickness measured using Freesurfer; v) vascular reactivity analysis [18]; vi) Resting-state fMRI analyses of the effect of stroke on functional brain networks. These measures will be compared between groups. Longitudinally, each patient will act as their own 'control' in linear regressions accounting for age, sex, initial stroke severity, lesion volume, and time. Finally, structural equation modelling will be used to

For peer review only - http://bmjopen.bmj.com/site/about/guidelines.xhtml

determine the relationship between demographics, comorbidities, blood and brain imaging biomarkers and cognitive and functional outcome measures.

Sample Size

The primary blood biomarker predictor is neurofilament light (NFL). Based on preliminary reported group differences [19], we expect 140 patients at baseline, and 60 patients at 3-months post stroke (when peak neurofilament light is expected to occur), will be able to detect group differences in neurofilament light levels between patients and controls (Wilcoxon Mann Whitney test, Cohens D=0.38 and 0.58 respectively, alpha=0.05, power=0.8, 1-tailed, allocation ratio 2:1). A much smaller sample size of 56 will be enough to detect correlations between NFL and time of sampling of the acute blood: (r^2=0.10 [19], alpha=0.05, power=0.8, 1-tailed).

The primary imaging outcome measure is white matter hyperintensity volume (WMHV). A sample size of 80 will be sufficiently powered to detect correlation between neurofilament light and white matter hyperintensity volume based on preliminary findings [25] (r=0.32, alpha 0.05, power 0.95). A sample size of N=70 will be sufficiently powered to detect relationship between peak plasma NFL levels and binary functional outcomes (based on similar effects observed after acute brain injury (R^2 = 0.28 [26], α=0.05, power=0.9). Expecting a 20-25% loss to follow-up rate (due to patient factors and technical factors), the stated sample size of 200 in blood biomarker sub-study is sufficiently powered to detect all the above-mentioned effects.

For peer review only - http://bmjopen.bmj.com/site/about/guidelines.xhtml

A secondary blood biomarker predictor is raised phosphorylated tau. Based on the mean and standard deviations reported for longitudinal change in phosphorylated tau (N=374) in population of elderly cognitively normal population [27], a sample size of N=65, will have 95% power in detecting any true change in mean over 1-year period (α=0.05, effect size 0.41). Thus, the stated sample size is sufficiently powered to detect longitudinal change in phosphorylated tau.

Discussion

Cognitive impairments are highly prevalent after stroke, having detrimental implications to recovery [1]. Identifying, monitoring and managing these deficits is consistently voted the highest priority by stakeholders [5]. Given the heterogeneity of stroke and its co-morbid conditions (such as aging, neurodegeneration and background small vessel disease), there is a need for large-scale longitudinal studies that enable accurate predictions of recovery and pave the way for personalised therapy allocation. Previous longitudinal studies of post-stroke outcomes have failed to identify reliable biomarkers of recovery [8]. On the one hand, this is due to a narrow focus on only limited contributors to recovery (e.g. lesion topology), despite the fact that stroke recovery is known to be inherently multi-modal [9]. On the other hand, this is due to using brief, shallow cognitive assessments in small-sized samples. Our main longitudinal study addresses this gap by coupling emerging blood and brain imaging

For peer review only - http://bmjopen.bmj.com/site/about/guidelines.xhtml

biomarkers with a novel scalable digital technology (IC3) that allows in-depth remote monitoring of cognition, thus providing a multi-modal account of recovery.

Moreover, current stand-alone cognitive screening tools commonly used in routine clinical care are either not stroke-specific, or not comprehensive. Commonly used tools, such as MOCA [15], Mini Mental State Examination (MMSE) [28], and Addenbrooke's Cognitive Examination-Revised (ACE-R) [29] are tailored to detect deficits in neurodegenerative dementias and are not suitable for patients with stroke, who often have domain-specific as well as domain-general deficits [10]. Additionally, increasingly used cognitive screens designed specifically for stroke, such as the Oxford Cognitive Screen (OCS), OCS-Plus and the Cognitive Assessment Scale for Stroke Patients (CASP) are not in-depth enough to allow a deep cognitive phenotyping and likely to miss the milder end of the severity spectrum. Assessments that are both stroke-specific and comprehensive, such as Birmingham Cognitive Screen (BCoS), are too resource-intensive for routine clinical practice or longitudinal research studies into post-stroke cognition. The IC3 assessment tool developed and applied in this study addresses these shortcomings, building the foundation for routine detailed monitoring of cognition and for scalable large-scale population-based studies of post-stroke cognitive impairment.

The blood biomarker sub-studies are expected to yield novel findings concerning the role of emerging biomarkers in neurodegenerative dementias, specifically amyloid and tau entities, as well as the role of biomarkers of neuroaxonal injury, such as

For peer review only

neurofilament light (NFL) and glial fibrillary acidic protein (GFAP), in determining cognitive outcomes post-stroke. The study will investigate the longitudinal dynamics of these biomarkers in the post-acute stroke period. The integration of both brain imaging biomarkers (e.g., white matter integrity, tissue microstructure, vascular reactivity, small vessel disease load) and blood biomarkers is expected to result in a cutting-edge and easily applicable predictive models for post-stroke cognition. The IC3 study is expected to make a significant contribution to a growing number of well-designed biomarker studies aimed at post-stroke cognition (such as R4VAD [30] and Discovery [31]) which have the potential to significantly advance the treatment of a disease that affects millions of people globally every year. These studies will enable more precise targeting of cognitive rehabilitation to individuals who are most at risk of progressive decline and those who have the highest potential for recovery.

Authors' Contributions

FG designed the study and obtained funding. DCG, FG, AH, HZ, WT, PH, SB and JK contributed to the data collection plan. FG, DCG, AH, WT and PH developed IC3 platform. DCG wrote the first draft of the manuscript. All authors reviewed and approved the manuscript.

Data management

For peer review only

All data collected will be pseudo-anonymised and securely stored on university servers. Blood samples will be stored at the Imperial College Clinical Research Facility and Imperial College Healthcare NHS Trust. Blood biomarkers will be processed and analysed following Good Laboratory Practice (GLP). Data management procedures will be compliant with both Imperial College London guidelines and GDPR regulations.

Ethics and Dissemination

Ethical considerations

This study has been approval by South West - Frenchay Research Ethics Committee (IRAS 299333), and authorized by the UK's Health Research Authority. All participants satisfying the inclusion and exclusion criteria will be approached and consented. If a patient is unable to provide fully informed consent, for example due to severe aphasia, we will assent with permission from a consultee. Patients that have been assented will be reconsented if they are subsequently able to provide full informed consent. Our study poses minimal risk to participants and will not affect their privacy, be invasive or restrictive. The consent procedure will be carried out in strict compliance with national legislation and General Data Protection Regulation.

Dissemination

The findings from this research will be disseminated through presentations at scientific conferences, as well as through publication in peer-reviewed journals. Additionally, the

For peer review only

data will be made available to authorized researchers to facilitate additional analysis, contributing to the development of multimodal biomarkers of recovery in stroke.

Funding statement

This research is funded by the UK Medical Research Council (MRC) under grant number MR/T001402/1.

Competing interests

PH is the co-founder and chief executive of H2 Cognitive Designs LTD, for which he receives remuneration. H.Z. has served at scientific advisory boards and/or as a consultant for Abbvie, Acumen, Alector, Alzinova, ALZPath, Annexon, Apellis, Artery Therapeutics, AZTherapies, Cognito Therapeutics, CogRx, Denali, Eisai, Nervgen, Novo Nordisk, Optoceutics, Passage Bio, Pinteon Therapeutics, Prothena, Red Abbey Labs, reMYND, Roche, Samumed, Siemens Healthineers, Triplet Therapeutics, and Wave, has given lectures in symposia sponsored by Cellectricon, Fujirebio, Alzecure, Biogen, and Roche, and is a co-founder of Brain Biomarker Solutions in Gothenburg AB (BBS).

For peer review only - http://bmjopen.bmj.com/site/about/guidelines.xhtml

References

[1] Douiri A, Rudd AG, Wolfe CD. Prevalence of poststroke cognitive impairment: South London stroke register 1995–2010. *Stroke* 2013; 44: 138–145.

[2] Grönberg A, Henriksson I, Stenman M, et al. Incidence of Aphasia in Ischemic Stroke. *Neuroepidemiology* 2022; 56: 174–182.

[3] Obaid M, Flach C, Marshall I, et al. Long-Term Outcomes in Stroke Patients with Cognitive Impairment: A Population-Based Study. *Geriatrics* 2020; 5: 32.

[4] Hill G, Regan S, Francis R, et al. Research priorities to improve stroke outcomes. *Lancet Neurol* 2022; 21: 312–313.

[5] Pollock A, St George B, Fenton M, et al. Top 10 Research Priorities Relating to Life after Stroke – Consensus from Stroke Survivors, Caregivers, and Health Professionals. *Int J Stroke* 2014; 9: 313–320.

[6] Quinn TJ, Richard E, Teuschl Y, et al. European Stroke Organisation and European Academy of Neurology joint guidelines on post-stroke cognitive impairment. *Eur Stroke J* 2021; 6: I–XXXVIII.

[7] Quinn TJ, Elliott E, Langhorne P. Cognitive and Mood Assessment Tools for Use in Stroke. *Stroke* 2018; 49: 483–490.

[8] Boyd LA, Hayward KS, Ward NS, et al. Biomarkers of stroke recovery: Consensus-based core recommendations from the Stroke Recovery and Rehabilitation Roundtable. *Int J Stroke* 2017; 12: 480–493.

[9] Simpkins AN, Janowski M, Oz HS, et al. Biomarker Application for Precision Medicine in Stroke. *Transl Stroke Res* 2020; 11: 615–627.

[10] Demeyere N, Riddoch MJ, Slavkova ED, et al. Domain-specific versus generalized cognitive screening in acute stroke. *J Neurol* 2016; 263: 306–315.

For peer review only - http://bmjopen.bmj.com/site/about/guidelines.xhtml

[11] Hampshire A, Hellyer PJ, Soreq E, et al. Associations between dimensions of behaviour, personality traits, and mental-health during the COVID-19 pandemic in the United Kingdom. *Nat Commun* 2021; 12: 4111.

[12] Krupp LB, LaRocca NG, Muir-Nash J, et al. The fatigue severity scale: application to patients with multiple sclerosis and systemic lupus erythematosus. *Arch Neurol* 1989; 46: 1121–1123.

[13] Herrmann N, Mittmann N, Silver IL, et al. A validation study of The Geriatric Depression Scale short form. *Int J Geriatr Psychiatry* 1996; 11: 457–460.

[14] Lawton MP, Brody EM. Assessment of older people: self-maintaining and instrumental activities of daily living. *The gerontologist* 1969; 9: 179–186.

[15] Nasreddine ZS, Phillips NA, Bédirian V, et al. The Montreal Cognitive Assessment, MoCA: A Brief Screening Tool For Mild Cognitive Impairment. *J Am Geriatr Soc* 2005; 53: 695–699.

[16] Friedman PJ. The Star Cancellation Test in acute stroke. *Clin Rehabil* 1992; 6: 23–30.

[17] Wardlaw JM, Smith EE, Biessels GJ, et al. Neuroimaging standards for research into small vessel disease and its contribution to ageing and neurodegeneration. *Lancet Neurol* 2013; 12: 822–838.

[18] Geranmayeh F, Wise RJS, Leech R, et al. Measuring vascular reactivity with breath-holds after stroke: A method to aid interpretation of group-level BOLD signal changes in longitudinal fMRI studies. *Hum Brain Mapp* 2015; 36: 1755–1771.

[19] Pedersen A, Stanne TM, Nilsson S, et al. Circulating neurofilament light in ischemic stroke: temporal profile and outcome prediction. *J Neurol* 2019; 266: 2796–2806.

[20] Chatterjee P, Doré V, Pedrini S, et al. Plasma Glial Fibrillary Acidic Protein Is Associated with 18F-SMBT-1 PET: Two Putative Astrocyte Reactivity Biomarkers for Alzheimer's Disease. *J Alzheimers Dis JAD* 2023; 92: 615–628.

For peer review only - http://bmjopen.bmj.com/site/about/guidelines.xhtml

[21] Gonzalez-Ortiz F, Turton M, Kac PR, et al. Brain-derived tau: a novel blood-based biomarker for Alzheimer's disease-type neurodegeneration. *Brain J Neurol* 2023; 146: 1152–1165.

[22] Zetterberg H, Bendlin BB. Biomarkers for Alzheimer's disease-preparing for a new era of disease-modifying therapies. *Mol Psychiatry* 2021; 26: 296–308.

[23] Wityk RJ, Pessin MS, Kaplan RF, et al. Serial assessment of acute stroke using the NIH Stroke Scale. *Stroke* 1994; 25: 362–365.

[24] van Swieten JC, Koudstaal PJ, Visser MC, et al. Interobserver agreement for the assessment of handicap in stroke patients. *Stroke* 1988; 19: 604–607.

[25] Gattringer T, Pinter D, Enzinger C, et al. Serum neurofilament light is sensitive to active cerebral small vessel disease. *Neurology* 2017; 89: 2108–2114.

[26] Graham NSN, Zimmerman KA, Moro F, et al. Axonal marker neurofilament light predicts long-term outcomes and progressive neurodegeneration after traumatic brain injury. *Sci Transl Med* 2021; 13: eabg9922.

[27] Moscoso A, Grothe MJ, Ashton NJ, et al. Longitudinal associations of blood phosphorylated Tau181 and neurofilament light chain with neurodegeneration in Alzheimer disease. *JAMA Neurol* 2021; 78: 396–406.

[28] Kurlowicz L, Wallace M. The mini-mental state examination (MMSE). *Journal of gerontological nursing* 1999; 25: 8–9.

[29] Mioshi E, Dawson K, Mitchell J, et al. The Addenbrooke's Cognitive Examination Revised (ACE-R): a brief cognitive test battery for dementia screening. *Int J Geriatr Psychiatry* 2006; 21: 1078–1085.

[30] Wardlaw JM, Doubal F, Brown R, et al. Rates, risks and routes to reduce vascular dementia (R4vad), a UK-wide multicentre prospective observational cohort study of cognition after stroke: Protocol. *Eur Stroke J* 2021; 6: 89–101.

[31] Rost NS, Meschia JF, Gottesman R, et al. Cognitive Impairment and Dementia After Stroke: Design and Rationale for the DISCOVERY Study. *Stroke*; 52.

For peer review only

For peer review only

Figures Legend

Figure 1– Infographic outlining the main specifications of the IC3 digital cognitive testing.

For peer review only - http://bmjopen.bmj.com/site/about/guidelines.xhtml

Figure 2 – Example of cognitive summary report.

Patient's performance (in purple) is outlined against a normative group (in grey), corrected for device, age, gender, and education. Each interval spacing represents 0.5 Standard Deviation units (SD) such that this participant's language comprehension performance was 1.5 SD below the mean normative performance. On the periphery of the spider diagram, the percentile score is shown for each task in purple, alongside a graded level of impairment.

Figure 3 – (A) Overview of the *main IC3* study and data collection. (B) Patient recruitment progress between February 2022 and March 2023.

Cognitive assessments will be performed using the IC3 online assessment. Additional sub-studies will include blood biomarkers and MRI brain imaging. The one-year follow-up is expected to take place between 12- and 24-months post-stroke.

For peer review only

For peer review only - http://bmjopen.bmj.com/site/about/guidelines.xhtml

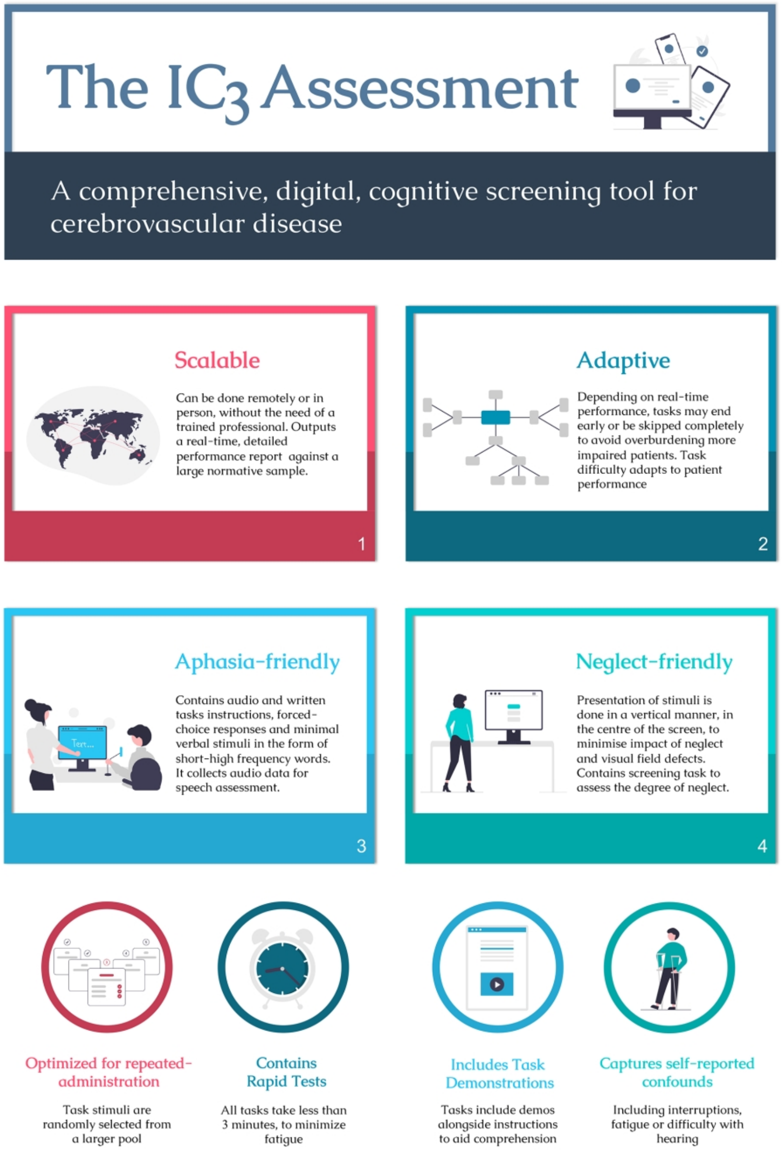

Figure 1– Infographic outlining the main specifications of the IC3 digital cognitive testing.

158x228mm (300 x 300 DPI)

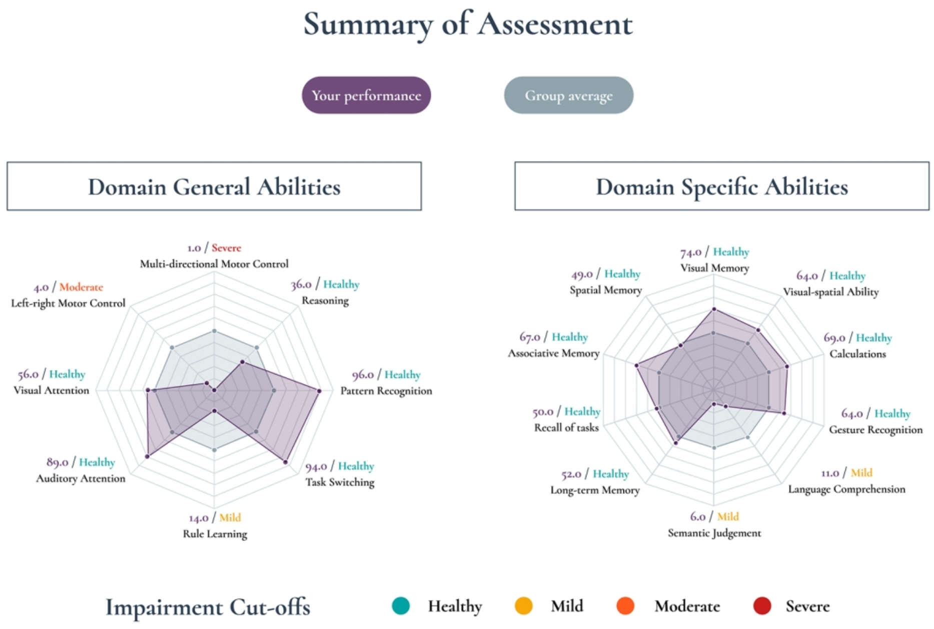

Figure 2 – Example of cognitive summary report.

150x106mm (300 x 300 DPI)

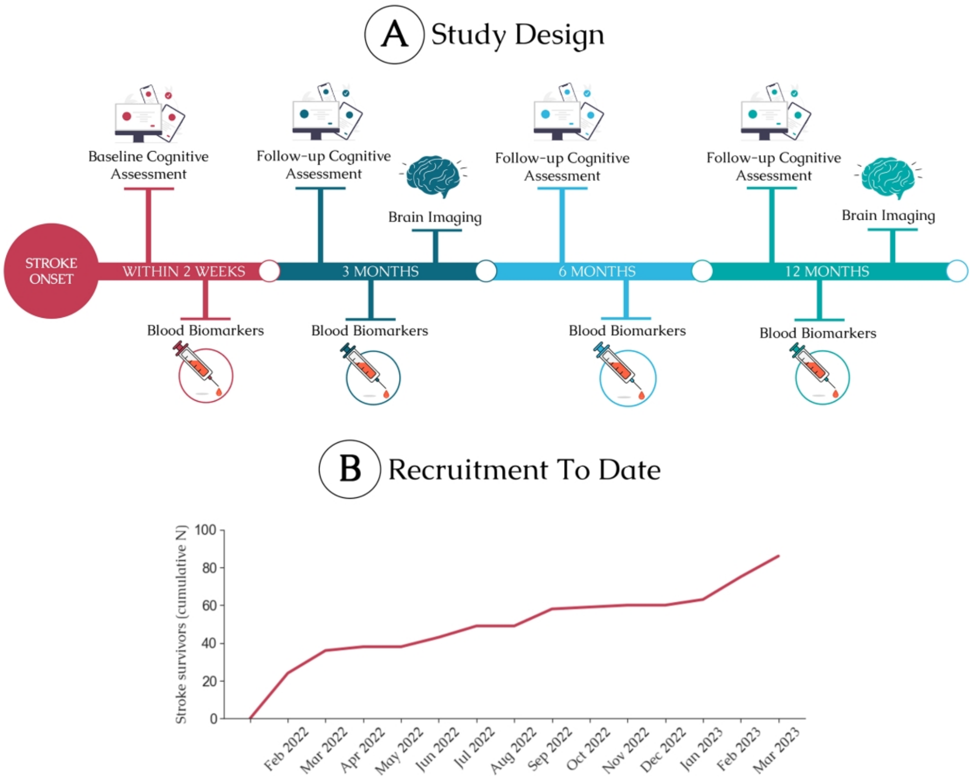

Figure 3 – (A) Overview of the main IC3 study. (B) Patient recruitment progress between February 2022 and March 2023.

188x156mm (300 x 300 DPI)

Supplementary Table 1 - Inclusion and Exclusion Criteria for Patients

Inclusion Criteria	***Exclusion Criteria***
○ Aged > 18<br>○ Evidence of confirmed stroke (not limited to first stroke)<br>○ Ability to concentrate for 15 minutes at a time to engage with cognitive testing	*For the main study:*<br>○ Pre-stroke diagnosis of dementia<br>○ Severe visuo-spatial problems<br>○ Fatigue limiting engagement in the tasks beyond 15 minutes<br>○ Active severe mental health diagnosis (e.g. severe depression or severe anxiety)<br>○ Severe hearing impairment in the presence of reading comprehension impairment resulting in inability to follow task instructions
	For the imaging sub-study:<br>○ Pregnancy<br>○ Ferromagnetic implants<br>○ Claustrophobia

For peer review only

Reporting checklist for cross sectional study.

Based on the STROBE cross sectional guidelines.

Instructions to authors

Complete this checklist by entering the page numbers from your manuscript where readers will find each of the items listed below.

Your article may not currently address all the items on the checklist. Please modify your text to include the missing information. If you are certain that an item does not apply, please write "n/a" and provide a short explanation.

Upload your completed checklist as an extra file when you submit to a journal.

In your methods section, say that you used the STROBE cross sectionalreporting guidelines, and cite them as:

von Elm E, Altman DG, Egger M, Pocock SJ, Gotzsche PC, Vandenbroucke JP. The Strengthening the Reporting of Observational Studies in Epidemiology (STROBE) Statement: guidelines for reporting observational studies.

		Reporting Item	Page Number
**Title and abstract**			
Title	#1a	Indicate the study’s design with a commonly used term in the title or the abstract	1
Abstract	#1b	Provide in the abstract an informative and balanced summary of what was done and what was found	1
**Introduction**			
Background / rationale	#2	Explain the scientific background and rationale for the investigation being reported	1
Objectives	#3	State specific objectives, including any prespecified hypotheses	4
**Methods**			
Study design	#4	Present key elements of study design early in the paper	8-9
Setting	#5	Describe the setting, locations, and relevant dates, including periods of	9-10

For peer review only

		recruitment, exposure, follow-up, and data collection	
Eligibility criteria	#6a	Give the eligibility criteria, and the sources and methods of selection of participants.	9
	#7	Clearly define all outcomes, exposures, predictors, potential confounders, and effect modifiers. Give diagnostic criteria, if applicable	10
Data sources / measurement	#8	For each variable of interest give sources of data and details of methods of assessment (measurement). Describe comparability of assessment methods if there is more than one group. Give information separately for for exposed and unexposed groups if applicable.	10-11
Bias	#9	Describe any efforts to address potential sources of bias	11-12
Study size	#10	Explain how the study size was arrived at	11-12
Quantitative variables	#11	Explain how quantitative variables were handled in the analyses. If applicable, describe which groupings were chosen, and why	10-11
Statistical methods	#12a	Describe all statistical methods, including those used to control for confounding	10-11
Statistical methods	#12b	Describe any methods used to examine subgroups and interactions	10-11
Statistical methods	#12c	Explain how missing data were addressed	n/a
Statistical methods	#12d	If applicable, describe analytical methods taking account of sampling strategy	n/a
Statistical methods	#12e	Describe any sensitivity analyses	8
**Results**			
Participants	#13a	Report numbers of individuals at each stage of study—eg numbers potentially eligible, examined for eligibility, confirmed eligible, included in the study, completing follow-up, and analysed. Give information separately for for exposed and unexposed groups if applicable.	n/a
Participants	#13b	Give reasons for non-participation at each stage	n/a
Participants	#13c	Consider use of a flow diagram	n/a

For peer review only

Descriptive data	#14a	Give characteristics of study participants (eg demographic, clinical, social) and information on exposures and potential confounders. Give information separately for exposed and unexposed groups if applicable.	n/a
Descriptive data	#14b	Indicate number of participants with missing data for each variable of interest	n/a
Outcome data	#15	Report numbers of outcome events or summary measures. Give information separately for exposed and unexposed groups if applicable.	n/a
Main results	#16a	Give unadjusted estimates and, if applicable, confounder-adjusted estimates and their precision (eg, 95% confidence interval). Make clear which confounders were adjusted for and why they were included	n/a
Main results	#16b	Report category boundaries when continuous variables were categorized	n/a
Main results	#16c	If relevant, consider translating estimates of relative risk into absolute risk for a meaningful time period	n/a
Other analyses	#17	Report other analyses done—e.g., analyses of subgroups and interactions, and sensitivity analyses	n/a
**Discussion**			
Key results	#18	Summarise key results with reference to study objectives	n/a
Limitations	#19	Discuss limitations of the study, taking into account sources of potential bias or imprecision. Discuss both direction and magnitude of any potential bias.	n/a
Interpretation	#20	Give a cautious overall interpretation considering objectives, limitations, multiplicity of analyses, results from similar studies, and other relevant evidence.	n/a
Generalisability	#21	Discuss the generalisability (external validity) of the study results	n/a
**Other Information**			
Funding	#22	Give the source of funding and the role of the funders for the present study and, if applicable, for the original study on which the present article is based	14

The STROBE checklist is distributed under the terms of the Creative Commons Attribution License CC-BY.
This checklist was completed on 07. June 2023 using https://www.goodreports.org/, a tool made by the EQUATOR Network in collaboration with Penelope.ai

For peer review only
